# Varicocele repair for severe oligoasthenoteratozoospermia: Scoping review of published guidelines, and systematic review of the literature

**DOI:** 10.1080/20905998.2024.2400629

**Published:** 2024-09-16

**Authors:** Andrian Japari, Walid El Ansari

**Affiliations:** a Fertility Clinic, Telogorejo Hospital, Semarang, Indonesia; b Department of Surgery, Hamad Medical Corporation, Doha, Qatar; c College of Medicine, Qatar University, Doha, Qatar; d Department of Clinical Population Health, Weill Cornell Medicine-Qatar, Doha, Qatar

**Keywords:** Male infertility, low sperm count, pregnancy rate, semen quality, systematic review, Varicocele

## Abstract

**Background:**

The outcomes of varicocele repair (VR) for severe oligozooasthenoteratozoospermia (OAT) have not been widely examined.

**Methods:**

Assessment of outcomes of VR after severe OAT, employing scoping review of published guidelines, and systematic review of literature. The Newcastle–Ottawa scale appraised the quality of included studies. Findings from both reviews were used to identify knowledge gaps and ways to enhance the evidence base.

**Results:**

No published guidelines exist specifically on VR for severe OAT. Of 731 articles retrieved, 15 were included, indicating a scarcity of studies appraising the topic. Most included studies exhibited high risk of bias and low-level evidence. Studies focused on basic sperm parameters; fewer examined hormonal/testicular volume changes, or pregnancy/live births. Studies suggested some post-VR sperm parameters improvements but mostly no changes in hormone levels/testicular volume. We identified four knowledge gaps: methodological issues; narrow scope of research and measurement aspects; lack of genetic considerations; and scarce economic/cost-effectiveness appraisals. We propose some precautions, remedies, and research questions to enhance the thin evidence base.

**Conclusions:**

VR for severe OAT has potential to improve sperm parameters. Scarcity of studies, high risk of bias, low-level evidence, and other limitations mitigate against drawing solid conclusions. Future research is required.

## Introduction

Varicocele is the most frequent correctable cause of infertility among men [1], with its prevalence estimated to be roughly 15% among the adult male general population [2,3], and about 35% and 80% in male partners of couples presenting with primary and secondary infertility, respectively [2–4]. Varicocele repair (VR), a management strategy for such condition, is accomplished via open surgical, laparoscopic, or radiological approaches [5–7]. However, controversies exist pertaining to the utility of VR in severe oligoasthenoteratozoospermia (OAT), with debate about its indications, non-indications, and potential outcomes. Severe OAT is a term used when sperm concentration is <5 million/ml, progressive motility is <32%, total sperm motility is <40%, and normal morphology is ≤4% [8]. The only systematic review on the topic included studies published up to 2020 [9].

On the one hand, some evidence points to potential improvements in semen parameters and fertility after VR in infertile men with impaired sperm parameters. Generally, among such men, sperm concentration and total count, motility, and normal morphology significantly improved after VR compared to controls with no VR [10]. Likewise, research and a systematic review found that VR for severe oligospermia can significantly improve semen parameters and fertility [9,11]. In addition, a meta-analysis of four retrospective studies (one on each of severe oligozoospermia, oligozoospermia, asthenoteratozoospermia, and oligoasthenozoospermia) of couples who had intra-cytoplasmic sperm injection (ICSI) with no prior vs. after VR demonstrated increases in pregnancy and live birth rates for those who had VR [12].

Conversely, other studies reported conflicting results, and several authors have voiced justifiable concerns. For instance, VR as a management strategy for patients with severe oligozoospermia remains deliberated [4,11,13]; and a meta-analysis assessed four randomized controlled trials of pregnancy after VR among oligozoospermic men to report a non-statistically significant moderately superior effect for VR compared to observation [14]. In addition, researchers have cautioned about concerns due to the ‘loss’ of precious time, lack of influence of sperm impairment on ICSI outcomes, and a higher risk of worsened sperm parameters post-VR. For instance, after VR for severe OAT, couples are likely to still require assisted reproductive technology (ART) to conceive, and a 3–6-month delay of ICSI while waiting for possible improvements in semen parameters is neither justified nor cost-effective [15,16]. This is important, particularly since a correlation study found no influence of impaired total sperm count, motility, and morphology on ICSI outcomes [17], questioning whether presumed semen improvements after VR in severe OAT are actually reflected in ICSI outcomes. Furthermore, patients with already altered spermatogenesis undergoing VR might be exposed to higher risk of deteriorating sperm parameters or even azoospermia after the procedure [4,18].

Given the above, little is known about the outcomes of VR in severe OAT. Therefore, the aim of the current report is to revisit the debate surrounding the topic, employing updated findings to furnish more clarity. The specific objectives were to: a) provide a general overview of the andrology-related benefits and potential risks of the VR procedure; b) undertake a scoping review to identify and describe the published guidelines on VR for severe OAT and appraise them for areas of full and partial agreement; and c) conduct a systematic review to identify the relevant published studies on VR for severe OAT and compare their sperm, hormonal, testicular volume, and fertility outcomes. In addition, we employed the emerging findings to detect any knowledge gaps and to generate an updated evidence-informed vision pertaining to VR for severe OAT, including research questions to enhance the evidence base, hence pushing forward the empirical, methodological, theoretical, clinical, and practical boundaries. Given the high prevalence of varicocele among men and its contribution to male factor infertility, particularly in severe OAT, employing VR as a treatment strategy requires a clearer understanding in order to enhance the outcomes related to sperm parameters, pregnancy rates, and live birth rates. These considerations inspired the concurrent scoping and systematic reviews, and the findings will be useful for practitioners, andrologists, urologists, in vitro fertilization (IVF) specialists, embryologists, policymakers, patients and couples, and other stakeholders.

## Materials and methods

### Scoping review of published guidelines

The aim of a scoping review is purposefully wide-ranging to explore and discover essential characteristics of a subject, uncover potential knowledge gaps, and exhibit critical examples. It is employed to ascertain the evidence on a topic and the critical attributes pertaining to a specified field [19]. Such reviews are valuable for far-reaching queries, e.g. ‘What information exists on the topic?’ and for gathering and appraising information [20]. Scoping reviews are critical when the information on a topic has not been meticulously examined, broad or complicated [21]. Hence, we selected this method to appraise the published guidelines of VR for severe OAT, a complex multifaceted topic. In line with Arksey and O’Malley [22], we employed a 5-step framework for scoping reviews that included: identifying the research question(s); identifying relevant studies; study selection; charting the data; and collating, summarizing, and reporting the results (Table 1). The review employed procedural and methodological rigor and clarity/transparency relating to this methodology as described by others [23,24].

**Table 1.** Five-step framework employed in the present scoping review for published guidelines.

Step	Description
Research questions	What is the current status of the published guidelines about VR for severe OAT?; What are the areas of full agreement between different guidelines?; What are the intersecting areas of partial agreement between different guidelines?; What areas are unique to each guideline? These queries were broken into features relating to varicocele status, sperm parameters, hormones, testicular volume and functional status, and others
Identifying relevant studies	Search strategies: structured search for published guidelines on VR for severe OAT using electronic databases (PubMed and Scopus), employing the appropriate search terms
Study selection	Published guidelines were selected when written by or on behalf of national or international andrology, urological, reproductive medicine, or male reproduction academy/ies association/s or society/ies
Charting the data	Data extracted consisted of items relevant to specific factors examined. Published guidelines were mapped employing the features described above
Collating, summarizing, and reporting results	Review team (AJ, WEA) assembled, grouped, synthesized and condensed the findings. Potential gaps were noted
Consultation exercise (optional)	Two experts specialized in the field reviewed the findings to provide opinion

Adapted from Arksey and O’Malley [22]; VR: varicocele repair; OAT: oligoasthenoteratozoospermia.

### Systematic review of the literature

This systematic review was undertaken and reported in accordance with the Preferred Reporting Items for Systematic Reviews and Meta-Analyses (PRISMA) statement [25–27], with additional guidance from the Cochrane Handbook of Systematic Reviews and Meta-Analyses for Interventions [28]. The purpose was to systematically review the evidence on VR outcomes among severe OAT patients. The protocol for this systematic review was not registered in PROSPERO.

### Research questions

We sought to answer two questions: what are the outcomes examined pertaining to the effects of VR among severe OAT patients; and, do these outcomes show improvement, deterioration, or no change after VR?

### Information sources and study selection

The search was performed on 31 January 2024 using PubMed and Scopus electronic databases and reference lists of included studies for articles published from 1 January 2000 through 31 January 2024. Two databases were searched to limit bias, as recommended by the Cochrane Collaboration [28]. In line with others [29], the search strategies were constructed from combinations of medical subject headings (MeSH) and keywords, further adjusted for the individual databases.

### Search strategy

For PubMed, the search terms were: ((‘severe oligo*’[All Fields] OR ‘extreme oligo*’[All Fields]) AND (‘varicocelectomies’[All Fields] OR ‘varicocelectomy’[All Fields] OR ‘varicocele repair’[All Fields] OR ‘sclero*’[All Fields] OR ‘emboli*’[All Fields])) AND (2000:2024[pdat]). For Scopus searches, we used the search terms: ALL (‘severe oligo*’ OR ‘extreme oligo*’) AND ALL (‘varicocele repair’ OR ‘sclero*’ OR ‘emboli*’ OR ‘varicocelectomy’) AND PUBYEAR > 1999 AND PUBYEAR < 2025.

The medical subject headings (MeSH) terms used were varicocele (All Fields) AND ‘low sperm count’ (MeSH Terms); varicocele (All Fields) AND ‘semen quality’ (MeSH Terms); varicocele (All Fields) AND ‘therapeutics’ (MeSH Terms); varicocele (All Fields) AND ‘fertile OR fertility’ (MeSH Terms); varicocele (All Fields) AND ‘infertile OR infertility’ (MeSH Terms); varicocele (All Fields) AND ‘reproduction OR reproductive’ (MeSH Terms).

In addition, the two authors (AJ, WEA) also conducted further searches using the reference lists of studies and review articles to select any relevant articles. The references to all included articles and relevant reviews were cross-checked.

### Data management

Results from each database were imported into Mendeley Desktop and duplications were removed. The authors (AJ, WEA) independently screened the titles and abstracts of the remaining studies for eligibility. Studies for which eligibility could not be gauged premised on the abstract were retrieved in full for further assessment. Titles and abstracts were screened, and the full text of relevant articles was subsequently reviewed before deciding on inclusion or exclusion. Data were then extracted, cross-checked and verified.

### Inclusion and exclusion criteria

The inclusion criteria were: (1) original studies; (2) any design; (3) published from 1 January 2000 through 31 January 2024; (4) assessed ‘VR’ and ‘male fertility/infertility’; and (5) included patients of any age and ethnicity. Only studies containing original research on possible association or cause-and-effect relationship between VR and sperm-, hormonal-, fertility-related parameters, or testicular volume were included. Exclusion criteria included review articles, editorials/correspondence, or commentaries, and studies that did not include VR, severe OAT, or any of the outcomes under examination.

### Data collection process and data items

Data was tabulated and described by WEA and AJ. A Microsoft Excel 2013 data extraction sheet was used to facilitate data collection. Extracted information included study design, sample size, semen parameters (sperm count, motility, morphology), hormonal parameters [follicle-stimulating hormone (FSH), luteinizing hormone (LH), inhibin B (IB), and total testosterone (TT)], testicular volume, and fertility-related outcomes (pregnancy rate, live birth rate).

**Figure 1.**
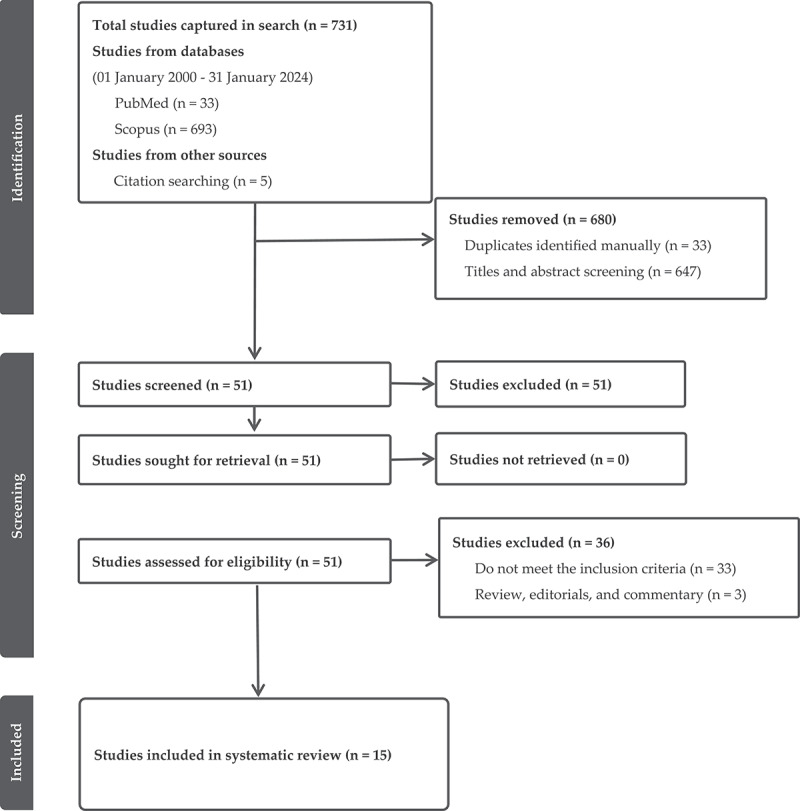
PRISMA flowchart of the search and screening results.

Figure 1 shows the PRISMA flowchart of the search and screening results. The initial search returned 731 articles (PubMed 33; Scopus 693; 5 citations from searching). We excluded 33 articles due to duplication and 647 articles after title and abstract screening. Of the remaining 51 articles, we further excluded 36 articles: 33 did not meet the inclusion criteria for further reading (including five that were not in English); three were review papers, editorials, and commentary. The remaining 15 studies were included in the current review.

### Assessment of quality

To assess the quality of the included studies, the present systematic review employed the Newcastle-Ottawa Scale [30]. The scale uses various items to rank the possibility of bias in three categories: selection, comparability, and outcome. Total scores of 7–9, 4–6, and 4 were classified as having a low, high, or very high risk of bias, respectively [30].

## Results

### Andrology-related effects of varicocele repair

Regardless of the method employed, the VR procedure to correct varicocele provides considerable fertility- and sexual-related potential benefits (Table 2). Nonetheless, the procedure is not without probable risks of recurrence, complications, or risks pertaining to testicular conditions, sperm parameters, oxidative stress, sperm DNA fragmentation, or antisperm antibodies.

**Table 2.** An overview of the andrology-related benefits and selected potential risks of the procedure of varicocele repair.

Benefits	Potential Risks
**Sexual Function**	
- *Improved*: sexual function [31]	
**Testicular Condition**	
- *Improved*: testicular volume [32]	- *Recurrence*: of varicocele [33]
- *Improved*: Sertoli cell function, spermatogenesis [34]	- *Complications*: Vascular compromise, testicular atrophy [33]; Hematoma, hydrocele [33]
**Sperm Parameters**	
- *Improved*: basic sperm parameters [9–11,13,18,34–44]	- *Decreased sperm count*: possibly up to azoospermia [4,18] - *Loss of valuable time*: Effect of VR may not be apparent until after 3-6 months due to duration of each spermatogenesis cycle, subject to age-related decline in female fertility/ovarian reserve. Hence, valuable time may sometimes be lost by delaying reproductive therapies [1,45]
**Sperm Retrieval**	
- *Increased*: sperm retrieval rate in azoospermia [46]	
**Oxidative Stress and Sperm DNA Fragmentation**	
- *Decreased*: OS and SDF [47,48] - *Decreased*: SDF [16,49] - *Decreased*: SDF prior to IVF, not IUI [50]	- *No improvement*: SDF rates in infertile patients with subclinical varicocele subjected to VR [51,52]
**Antisperm Antibody**	
- *Decreased* [53]	- *Increased* [53–55]
**Hormones**	
- *Normalized/Improved*: testosterone levels, especially in men with clinically palpable varicoceles + abnormal semen parameters [56–61]	
- *Increased*: inhibin B [34]	
- *Decreased*: follicle stimulating hormone [34]	
**Pregnancy and Birth**	
- *Improved*: pregnancy rate [5,42,46,62,63]	*No impact*: on pregnancy rate and miscarriage rate [64]
- *Improved*: pregnancy and live birth rates [16,49,50]	

OS: Oxidative stress; SDF: sperm DNA fragmentation; IUI: Intrauterine insemination; IVF: In vitro fertilization.

### Varicocele repair for oligoasthenoteratozoospermia: Published guidelines

This review identified and analyzed four international guidelines with recommendations pertaining VR for OAT (Table 3). The guidelines were developed, individually or in combination, by prominent agencies, including the European Academy of Andrology (EAA), European Association of Urology (EAU), American Urological Association (AUA), American Society for Reproductive Medicine (ASRM), and Society for Male Reproduction and Urology (SMRU). The Table depicts brief descriptions of each guideline in terms of the clinical and laboratory scenarios where VR for male infertility, specifically OAT, is recommended, considered, or not indicated.

**Table 3.** Published guidelines on VR for OAT.

Guideline and Brief Description
***EAA Guideline Management of OAT*** [65]
- *Recommended*: VR in young males with progressive testicular failure and/or seminal deterioration
- C*an be discussed*: VR for infertile couples reporting OAT associated with palpable varicocoele
- *Only monitor*: cases with subclinical varicocele
***AUA/ASRM******Diagnosis and Treatment of Infertility in Men: Guideline part I and part II*** [66,67]
- *Consider*: VR among men attempting to conceive, who have palpable varicocele(s), infertility, abnormal semen parameters, except for azoospermic men
- *Not recommended*: VR in men with nonpalpable varicoceles detected solely by imaging
- *Absence of definitive evidence*: VR prior to ART for clinical varicocele in NOA patients
***ASRM/SMRU Report on Varicocele and Infertility: A Committee Opinion*****(2014)** [45]
- *Consider*: VR when most/all of these criteria are met: 1) varicocele is palpable on physical examination of scrotum; 2) couple has known infertility; 3) female partner has normal fertility or potentially treatable cause of infertility, and time to conception is no concern; 4) male partner has abnormal semen parameters
- *Consider*: VR in adolescents/young males with varicoceles + reduced ipsilateral testicular volume
- *Not indicated*: VR for patients with either normal semen quality, isolated teratozoospermia, or subclinical varicocele
***EAU Guidelines on Sexual and Reproductive Health*****(2023)** [51]
- *Recommended in adolescents*: VR for those with ipsilateral reduction in testicular volume + evidence of progressive testicular dysfunction
- *Recommended in infertile men*: VR for clinical varicocele + abnormal semen parameters + otherwise unexplained infertility in couple where female partner has good ovarian reserve to improve fertility rates
- *Consider*: VR for men with raised DNA fragmentation with otherwise unexplained infertility, or who have had failed ARTs, including RPL, failure of embryogenesis, and implantation
- *Not indicated*: VR for infertile men with normal semen analysis and men with sub-clinical varicocele

ASRM: American Society for Reproductive Medicine; AUA: American Urological Association; EAA: European Academy of Andrology; EAU: European Association of Urology; SMRU: Society for Male Reproduction and Urology; ART: Assisted Reproductive Technology; FSH: Follicle stimulating hormone; IVF: In vitro fertilization; NOA: non-obstructive azoospermia; OAT: Oligoasthenoteratozoospermia; RPL: Recurrent Pregnancy Loss; VR: varicocele repair.

**Figure 2.**
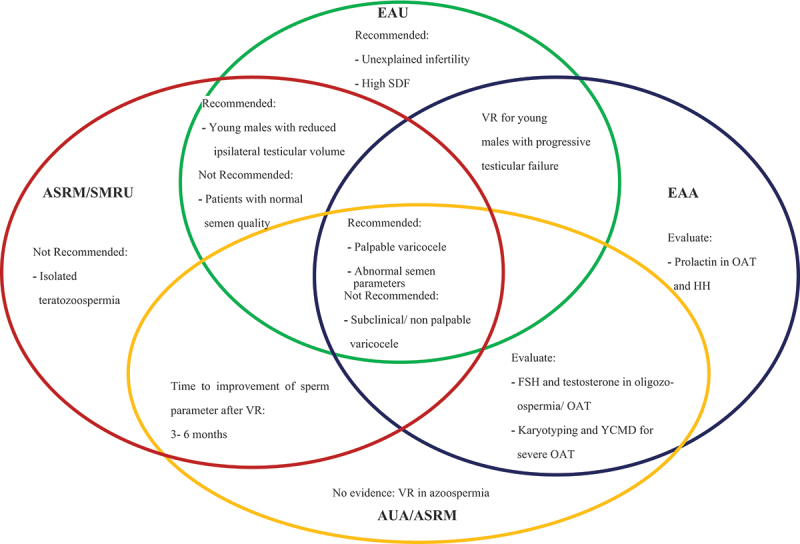
Venn diagram of full and partial agreements of current four published guidelines on VR for OAT.

Note: ASRM/SMRU: Practice Committee of American Society for Reproductive Medicine and Society for Male Reproduction and Urology; EAA: European Academy of Andrology; EAU: European Association of Urology; AUA/ASRM: American Urological Association/American Society of Reproductive Medicine; HH: Hypogonadotropic Hypogonadism; OAT: Oligoasthenoteratozoospermia; SDF: sperm DNA fragmentation; VR: varicocele repair; YCMD: Y chromosome microdeletion

Figure 2 depicts a Venn diagram of the retrieved current guidelines of VR for OAT that were identified, outlining the areas unique to each guideline as well as the intersecting areas of full and partial agreements between different guidelines. Areas of full agreement (consensus) include the recommendation for VR for palpable varicocele with abnormal semen parameters and the non-indication of VR for subclinical or non-palpable varicocele. On the other hand, areas of partial agreement included issues related to the indication of VR for young people with progressive testicular failure or with reduced ipsilateral testicular volume; the need for hormonal evaluations for particular patients; the time needed for improvement of sperm parameters after VR; and the non-indication of VR for normal semen quality.

### Varicocele repair for severe oligoasthenoteratozoospermia: Published studies

The assessment of quality of the 15 included studies in the current review (Table 4) showed that most studies exhibited a high risk of bias except for two studies that had low risk [34,42].

**Table 4.** Assessment of quality of included studies^*a*^.

Study	Selection	Comparability	Outcome	Total	Risk of Bias
Poulakis 2006 [35]	* * *	—	* * *	6	High
Ishikawa 2008 [36]	* * *	—	* * *	6	High
Smit 2010 [37]	* * *	—	* * *	6	High
Ghanem 2011 [34]^*b*^	* * *	*	* * *	7	Low
Leung 2013 [38]	* * *	—	* * *	6	High
Enatsu 2014 [13]	* * *	—	* * *	6	High
Dubin 2018 [11]	* * *	—	* * *	6	High
Gupta 2018 [39]	* * *	—	* * *	6	High
Almekaty 2019 [40]	* * *	—	* * *	6	High
Turgut 2020 [42]^*a*^	* * *	*	* * *	7	Low
Addar 2021 [18]	* * *	—	* * *	6	High
Majzoub 2021 [9]	* * *	—	* * *	6	High
Vu Tan 2023 [43]	* * *	—	* * *	6	High
Alkhayal 2024 [44]	* * *	—	* * *	6	High

Due to space considerations, only the first author is cited; ^*a*^The Newcastle Ottawa Scale [30]; ^*b*^Retrospective design with controls; One study [41] was not included in the assessment of quality as it was a case report.

Table 5 depicts the included studies, the design and level of evidence, as well as the parameters examined by each study. The predominant study design was of retrospective nature (*n* = 10, 66.7%), and the level of evidence of most studies (80%) was not high (level III).

**Table 5.** Included studies of VR for severe OAT: design, levels of evidence, and four sets of outcomes*.

Study	Design (LoE)	Sperm Outcomes			Hormonal Outcomes					Fertility Outcomes	
		Count	Motility	Morph	FSH	LH	IB	TT	TV	PR	LBR
Poulakis 2006 [35]^*a*^	Rp (III)	+	+	+				+		T 33.3%; Sp 21.2%; ART 12.1%	T 33.3%; Sp 21.2%; ART 12.1%
Ishikawa 2008 [36]^*b*^	Case-control (III)	+	=		=	=		=	=	Sp 7.4%	
Smit 2010 [37]^*c*^	Rp (III)	T +	T +	T =	R =, NR—	R =, NR —	=	=		Sp 37%; ART 22%	
Ghanem 2011 [34]^*d*^	Rp (III)	+	+	+	+		+	=	=	Sp 15% (9/59); ICSI 32.3% (10/31); IVF 22.2% (2/9); IUI 14.3(1/7)%	Sp 15%; ICSI 29%; IVF 22%; IUI 14%
Leung 2013 [38]^*e*^	Case series (IV)	+	+	+						T 75% (12/16); Sp 41.7% (5/12); IUI 8.3%% (1/12); IVF 50% (6/12)	
Enatsu 2014 [13]^*f*^	Rp (III)	T +, R +, NR =	T +, R +, NR =		=	=		=		Sp: T 17.6% (18/102); R 15.6% (16/102); NR 2% (2/102)	
Dubin 2018 [11]^*g*^	Rp (III)	+	+							Sp 10% (1/10); IUI 28.6% (2/7)	
Gupta 2018 [39]^*h*^	Rp (III)	+	+							Sp 37.1%	
Almekaty 2019 [40]^*i*^	Prospective randomized study (II)	G1 + G2 +	G1 + G2 +						G1 +, G2 =	Sp. G1 40%; G2 30%	
Najari 2019 [41]^*j*^	Case Report (V)	**+**	**+**	**+**				**+**		ART 100%	ART 100%
Turgut 2020 [42]^*j*^	Rp (III)	**+**	**+**	**+**						Sp 13.4%; ART 24.9%	
Addar 2021 [18]	Rp (III)	**+**^*k*^	—								
Majzoub 2021 [9]^*l*^	Rp (III)	**+**	**+**	**=**	**=**	**=**		**=**			
Vu Tan 2023 [43]	Prospective non controlled (III)	**+**	**+**	**+**^*m*^						Sp 100%	
Alkhayal 2024 [44]^*n*^	Rp (III)	**+**	**=**	**=**						100% (5/5)^*o*^	20% (1/5) ^*p*^

Due to space considerations, only the first author is cited; empty cells denote data not available/cannot be extracted; *Levels of evidence grading based on Dang et al. (2021) [68] and Clinical Information Access Portal (Undated, c2018) [69]; **+**: significant improvement (*p* < 0.05); **=**: no significant change (*p* > 0.05); —: significant deterioration (*p* < 0.05); ART: Assisted reproductive technology; FSH: Follicle stimulating hormone; G: Group; IB: Inhibin B; ICSI: Intracytoplasmic sperm injection; IUI; Intrauterine insemination; IVF: In vitro fertilization; LBR: Live birth rate; LH: Luteinizing hormone; Morph: morphology; OAT: Oligoasthenoteratozoospermia; PR: Pregnancy rate; Rp: Retrospective; Sp: Spontaneous pregnancy; T: Total; TT: Testosterone; TV: Testicular volume; Only those with sperm concentration < 5 million/ml included in the table representing: ^*a*^70.2%, ^*b*^90%, ^*e*^36.4%, ^*h*^62.5%, ^*n*^38.5% of the sample; ^*c*^R (responders) comprised 63.2% of the sample, NR (non-responders) comprised 36.8% of sample; ^*d*^ Retrospective design with controls; ^*f*^R (responders), 41.1% of sample; NR (non-responders), 58.9% of sample; ^*g*^Prospectively collected data, no controls; ^*i*^G1: artery-preserving varicocelectomy (49.7% of sample), G2: artery ligation varicocelectomy (50.3% of sample); ^*j*^Study did not provide *p* value, significance could not be determined; ^*k*^8.9% of sample showed deterioration; ^*l*^ Comprises original study and meta-analysis, only the findings of original study included in the table; ^*m*^Percentage of improvement cannot be computed as initial value was zero; ^*o*^Not reported whether pregnancies were spontaneous or assisted; ^*p*^ Not reported whether live births were after spontaneous or assisted pregnancies.

Some interesting trends emerged: a) In terms of sperm parameters, all studies (*n* = 15) assessed the VR outcomes pertaining to sperm count, motility, but fewer studies (*n* = 9, 60%) assessed the morphology; b) for hormones (FSH, IB, LH, TT), only one study (6.7%) [37] evaluated all four hormonal-related outcomes, while three studies (20%) did not assess any [38,39,44]. The least evaluated hormonal outcome was IB, appraised by only two studies (13.3%) [34,37], and the most common was TT, examined by seven studies (46.7%) [9,13,34–37,41]; c) regarding changes in testicular volume, only three studies (16.7%) evaluated it [34,36,40]; d) pertaining to fertility-related outcomes, 13 studies (86.7%) assessed pregnancy rates [11,13,34–44], while only four studies (26.7%) examined live birth rates [34,35,41,44]; and, e) for the full range of the 10 VR-related outcomes, no studies appraised the full range of such outcomes. One study appraised nine parameters [34], while the remaining studies evaluated various numbers of parameters, ranging from two [18] to eight outcomes [37].

Table 6 depicts a summary of the improvement, deterioration, or no improvement of the sperm and hormonal, and testicular volume parameters, as well as a narrative pertaining to the fertility outcomes after VR for severe OAT. At times, the outcomes of some parameters were bidirectional (sperm morphology, FSH, IB, and TT) e.g. some participants showing improvements while others exhibiting no change; or even multi-directional (sperm motility), where some participants showed improvements, while others had deterioration or no change.

**Table 6.** VR for severe OAT: summary of improvement, deterioration, or no change of parameters.

Parameter	N	Improved	Deteriorated	No significant change
**Sperm**				
Count	15/15	15/15 studies (100%) − 1 study [18], despite improvement in majority of the sample, 8.9% deteriorated) − 1 study [13], despite improvement across total sample, no significant change among 58.9% (NR)	0/15 studies (0%)	0/15 studies (0%)
Motility	15/15	12/15 studies (80%) − 1 study [13], despite improvement across total sample, no significant change among 58.9% (NR)	1/15 studies (6.7%) [18]	2/15 studies (13.3%) [36,44]
Morphology	9/15	6/9 studies (66.7%) [34,35,38,41–43]	0/9 studies (0%)	3/9 studies (33.3%) [36,37,44]
**Hormonal**				
FSH	5/15	1/5 studies (20%) [34]	0/5 studies (0%)	4/5 studies (80%) [9,13,36,37] − 1 study [37], despite no significant change in majority of sample, 36.8% (NR) deteriorated
LH	4/15	0/4 studies (0%)	0/4 studies (0%)	4/4 studies (100%) [9,13,36,37] − 1 study [37], despite no significant change in majority of sample, 36.8% (NR) deteriorated
IB	2/15	1/2 studies (50%) [34]	0/2 studies (0%)	1/2 studies (50%) [37]
TT	7/15	2/7 studies (28.6%) [35,41]	0/7 studies (0%)	5/7 studies (71.4%) [9,13,34,36,37]
**Testicular volume**	3/15	0/3 studies (0%)	0/3 studies (0%)	3/3 studies (100%) [34,36,40] − 1 study [40], despite no significant change in majority of sample, 49.7% ^*a*^ showed improvement
**Fertility Outcomes**				
Pregnancy rate	13/15	13 studies (86.7%): 2 did not report parameter; 1 reported it, but did not indicate whether pregnancies were spontaneous/assisted; 5 reported spontaneous pregnancies only; 1 reported assisted pregnancies only; 6 reported both		
Live birth rate	4/15	4 studies (26.7%): 11 did not report parameter; 1 reported it, but did not indicate whether given pregnancies were spontaneous/assisted; 1 reported that pregnancies were assisted; 2 reported that pregnancies were both spontaneous and assisted		

N: number of studies that undertook or reported this parameter of the total studies; FSH: Follicle stimulating hormone; IB: Inhibin B; LH: Luteinizing hormone; NR: non-responders; TT: Testosterone; VR: varicocelectomy; ^*a*^ Artery-preserving varicocelectomy group.

Generally, most studies showed improvements in sperm count (*n* = 15/15), motility (*n* = 12/15), and morphology (*n* = 6/9), with fewer reporting no significant change in motility (*n* = 2/15) and morphology (*n* = 3/9), and one observing deterioration of sperm motility [18]. In terms of hormonal outcomes, fewer studies (range = 13.3%–46.7%) reported no significant change of FSH, LH, IB or TT; although one study showed FSH and IB improvements [34]; and two studies noted TT improvement [35,41]. Particularly for FSH, a study found that despite no improvement in majority of the sample, 36.8% of the sample deteriorated [37]. Similarly, pertaining to LH, the same study found that despite no improvement in majority of the sample, 36.8% of the sample deteriorated [37]. No other deteriorations were observed across the hormonal outcomes. As for testicular volume, the three studies that assessed it observed no improvement [34,36,40], although one of these studies [40] found that despite no significant change in majority of sample, 49.7% participant in artery-preserving varicocelectomy group showed improved testicular volume.

Table 7 combines three aspects, namely, the summary of changes of each domain of the parameters under examination after VR, the number of studies contributing to such changes, as well as their levels of evidence. Several points stand out: a) the literature exhibits few studies that addressed the outcomes of VR for severe OAT; b) in most cases, there was low-level evidence or not sufficient evidence; and c) whilst some parameters exhibited some improvements, non-improvement was not a rare observation, and occasionally some deterioration in some of the parameters was observed across some subsets of patients. Collectively, these characteristics mitigated against the ability to draw solid conclusions for the relationships and behaviors of some of the parameters after VR for severe OAT.

**Table 7.** Summary of changes in fertility parameters after VR for severe OAT and their levels of evidence*.

		Changes in parameter after VR			Summary				
					Improved		Deteriorated/No significant change		
Parameter		Improved	Deteriorated	No significant change	Low LoE	High LoE	Low LoE	High LoE	Suggested Verdict ^*a*^
**Sperm**									
Count	Studies (n)	15^*b, c*^	0	0	15		0		
	LoE	1 L II, 12 L III, 1 L IV, 1 L V	—	—	14	1	0	0	All improved, but mostly low-level evidence, with only one high-level evidence study
Motility	Studies (n)	12^*c*^	1	2	12		3		
	LoE	1 L II, 9 L III, 1 L IV, 1 L V	1 L III	2 L III	11	1	3	0	Mostly improved, with one high-level evidence study; sometimes no significant change, rarely deteriorated, but low-level evidence
Morph	Studies (n)	6	0	3	6		3		
	LoE	4 L III, 2 L IV	—	3 L III	6	0	3	0	Two thirds improved, one third not improved, but low-level evidence
**Hormonal**									
FSH	Studies (n)	1	0	4 ^*d*^	1		4		
	LoE	1 L III	—	4 L III	1	0	4	0	Mostly no significant change, sometimes improved, but low-level evidence
LH	Studies (n)	0	0	4^*d*^	0		4		
	LoE	—	—	4 L III	0	0	4	0	All no significant change, but low-level evidence
IB	Studies (n)	1	0	1	1		1		
	LoE	1 L III	—	1 L III	1	0	1	0	Insufficient evidence, low-level evidence suggests equivocal
TT	Studies (n)	2	0	5	2		5		
	LoE	1 L III, 1 L IV	—	5 L III	2	0	5	0	Mostly no significant change, sometimes improved, but low-level evidence
**Testicular**	Studies (n)	0	0	3^*e*^	0		3		
**volume**	LoE	—	—	3 L III	0	0	3	0	All no significant changes, but low-level evidence
**Fertility Outcomes**									
PR	Studies (n)	13							
	LoE	1 L II,10 L III, 1 L IV, 1 L V; All studies that measured this outcome reported either spontaneous or assisted pregnancies							
LBR	Studies (n)	4							
	LoE	3 L III, 1 L V; All studies that measured this outcome reported live births							

*Levels of evidence grading based on Dang et al. (2021) [68] and Clinical Information Access Portal (Undated, c2018) [69], evidence level III or more are considered low, level II and less considered high; —: not applicable; FSH: Follicle stimulating hormone; IB: Inhibin B; L: Level; LBR: Live birth rate; LH: Luteinizing hormone; LoE: level of evidence; Morph: morphology; PR: Pregnancy rate; TT: Testosterone; ^*a*^Suggested verdict based only on studies that measured the given outcome, verdict could change if all studies had assessed the given outcome; ^*b*^Despite improvement in majority of sample, 8.9% deteriorated [18]; ^*c*^Despite improvement across total sample, no significant change among 58.9% (non-responders) [13]; ^*d*^Despite no significant change in majority of sample, 36.8% (non-responders) deteriorated [37]; ^*e*^Despite no significant change across majority of sample, 49.7% (artery-preserving varicocelectomy group) showed improvement [40].

### Now what? knowledge gaps and way forward to enhance the evidence base

The analysis of the guidelines and the available relevant literature revealed several knowledge gaps (Table 8). Section A of the Table depicts that these gaps could be broadly classified into a wide range of research-related methodological limitations that included the scarce research on the topic, study designs, scale of research, sample sizes, ultimate endpoint/s, and confounding; as well as issues having to do with narrow scope research, measurement aspects and multi-directional effects, to include issues of non-reporting and technical aspects of the measurement of hormones etc., lack of consideration of variability of measurements, lack of serial follow ups, multi-directional effects, and the non-assessments of some hormones and/or testicular volume in some studies. Other knowledge gaps included insufficient attention to genetic considerations, and the dearth of data on cost-effectiveness of the VR procedure compared to other methods of management of severe OAT.

**Table 8.** VR for severe OAT: knowledge gaps and potential way forward to enhance the evidence base.

A) Knowledge gap	Description	B) Way forward: Enhancing the evidence base
Methodological		
Insufficient research	Fifteen studies were identified and included in this systematic review	Evidence base of effectiveness is currently rather thin. Research required to enhance the evidence base of the indications for, patient selection and effectiveness and outcomes of VR in severe OAT
Level of evidence/Study design	Majority of included studies (*n* = 10, 66.7%) were retrospective, another 2 studies (13.3%) were a case report [41] and a case series [38], all representing low level evidence; only 1 (6.7%) prospective randomized study representing high level evidence	Need for more high-quality prospective study designs, as well as meta-analyses of prospective studies to enhance the evidence; when prospective studies are undertaken, where feasible, they need to include comparator or control group, and where feasible not self-comparison, if research can ensure that it is not exposing subjects to harmful risk factors as this is unethical [70]
Scale of research	All identified studies appear to be single center studies. Larger studies are associated with less random error; smaller studies are prone to greater random error [71]	Single center studies have merits; but multi-center are needed as generally they are bigger, more powerful, advantageous, with quicker recruitment, diverse population, increased generalizability and better clinical decision making [72,73], but need to satisfy technical/interpretative integrity
Sample size	All studies had modest sample sizes, might fail to detect an effect if it existed (Type II error), except one study (6.7%) with larger sample (*n* = 302) [40]	Relatively small sample sizes limit statistical power for identifying true associations. Enhance evidence by: - More studies with larger sample sizes to be able to detect and gain in-depth understanding of effects should they exist
Ultimate endpoint/s	Only four studies (26.7%) examined the live birth rate after VR as an ultimate outcome [37,41–43]	Studies should aim to measure ultimate outcome of sperm improvement reflected by live birth rate [9], as improved pregnancy rates do not necessarily translate into live births. Take home baby is the goal of infertile couples [74]. Related queries for VR in severe OAT include: - Is there consensus about spontaneous pregnancy rate? If yes, what is the rate? - If not, then consensus required on the live birth rate
Confounding	Almost all studies (93.3%) did not simultaneously account for grade of varicocele while analyzing outcomes of VR, except one study [40]	Generally, higher grade varicocele, if repaired, has better outcomes than lower grade varicocele [75–77]. Related queries for VR in severe OAT include: - What is the effectiveness of VR stratified by varicocele grade? - Does repair of higher-grade varicocele generate superior outcomes than lower grade varicocele? - Which varicocele grade is likely to benefit most from VR in severe OAT? - Is grade of varicocele predictive of outcomes?
Narrow scope research + measurement aspects + multi-directional effects		
Basic semen parameters		
Technical aspects	Technical approaches: Semen analysis undertaken using WHO 1992 [36], WHO 1999 [34,35,37,39]; or using WHO 2010 [9,40,43]; one study (6.7%) did not report the WHO guideline version used [13]	- Ensure reporting of how measurement was undertaken - Ensure uniformity in measurement technique for consistently comparable data across studies/countries
Lack of consideration of variability	Six studies (40%) did not specify whether single or replicate semen analyses were undertaken [9,18,35,37,38,44]; does not account for biological and intra/inter observer variabilities for all and infertile men [78]	- Routine assessment of two replicates of semen samples required for confirmation, accuracy and reliability and to account for potential patient- or lab-related variations [44,78]
Lack of serial follow ups	One third of studies (33.3%) did not undertake serial follow ups of semen analysis after VR to confirm any improvements in semen parameters observed [9,18,36,37,41]	Only two third studies (66.7%) undertook serial semen analysis after VR. For studies that examine semen analysis after VR, serial (≥2 times over at least 3 months to 1 year) needed for verification/confirmation, and for attesting maintenance/sustainability of any observed effects [18]
Motility: Tridirectional effects	Most studies (80%) reported improved sperm motility, two (13.3%) observed no significant change, and one (6.7%) showed deterioration	- What are the characteristics of patients who are likely to show improvement, no significant change, or even deterioration of sperm motility after VR in terms of age, varicocele grade, smoking habits, patients’ occupation, or associated comorbidities? - Which of these or other characteristics, if any, exerts stronger effects on motility and can hence be potentially used to predict this outcome?
Morphology: Bidirectional effects	Most studies (66.7%) exhibited improved sperm morphology, three (33.3%) observed no significant change	- What are the patient attributes associated with improvement, no significant change, or even deterioration of sperm morphology after VR in terms of age, body mass index, varicocele grade, smoking habits, or associated comorbidities? - Which of these or other attributes, if any, exerts stronger effects on morphology and can hence be used to predict this outcome?
Hormones		
FSH		
Non-reporting	One fifth of studies (*n* = 3) did not examine any FSH level as marker of testicular function [38,39,44]; seven studies (46.7%) assessed only pre-VR FSH	Normal secretion of FSH is required for normal spermatogenesis; need to consistently assess pre-/post-FSH level as testicular function marker [79–81], despite that one study found that pre-VR serum FSH levels was unrelated to post-VR semen parameters and pregnancy (Poulakis et al. 2010). Related queries include: - What are the relationships between FSH level and sperm parameters? - What are the relationships between FSH level and pregnancy? - How accurate can FSH level reflect deteriorated sperm parameters before VR? - Can VR outcomes be predicted by pre-VR FSH level? If yes, what cutoff levels predict favorable outcomes?
Bidirectional effects	Most studies (80%) exhibited no change in FSH after VR, one (*n* = 1, 20%) observed improvements	- What are the age, varicocele grade, testicular volume, testicular blood flow, and genetic characteristics of patients who are likely to have improved FSH levels after VR? - Which of these or other characteristics, if any, exerts stronger effects on FSH and can thus be used to predict this hormonal outcome?
Technical aspects	Technical approaches: 75% of studies that measured FSH did not report how it was measured. FSH might have been measured by different methods	Future studies should: − Ensure reporting of how measurement was conducted - Ensure uniformity in measurement technique for consistently comparable data across studies/countries
LH		
Non-reporting	40% studies did not examine any LH levels as testicular function marker [34,35,38,39,41,44]; five studies (33.3%) assessed only pre-VR LH level [11,18,40,42,43]	Normal secretion of LH is required for normal spermatogenesis [80,81]; LH level should be obtained if sperm concentration is severely impaired for evaluation of causes of primary hypogonadism [80]. Related queries for VR in severe OAT include: - What are the relationships between LH level and sperm parameters? - What are the relationships between LH level and pregnancy? - How accurate can LH level reflect deteriorated sperm parameters before VR? - Can VR outcomes be predicted by pre-VR LH level? If yes, what cutoff levels predict favourable outcomes?
Technical aspects	Technical approaches: 77.8% studies that measured LH did not report how it was measured [11,13,18,37,40,42,43]. LH might have been measured by different methods	Future studies should: − Ensure reporting of how measurement was undertaken - Ensure uniformity in measurement technique for consistently comparable data across studies/countries
Inhibin B		
Non-reporting	Almost all studies (86.7%) did not examine IB level as testicular function marker, except two studies that measured pre-/post- VR IB [34,37]	- Inhibin B is considered novel assessment indicator of testicular function, specifically spermatogenesis [82]; has significant positive correlation with sperm parameters and testicular volume in varicocele [83–85] - Prospective studies needed to assess IB levels to: determine precisely its relationship to the extent of testicular damage; and evaluate utility of its pre-VR as a predictor of improvement in sperm parameters, and its cutoffs
Bidirectional effects	Half the studies that assessed IB exhibited no change in IB after VR; the other half observed improvements	- What are the age, varicocele grade, testicular volume, testicular blood flow, and genetic characteristics of patients who are likely to have improved IB levels after VR? - Which of these or other characteristics, if any, exerts stronger effects on IB and can hence be used to predict this outcome?
Technical aspects	Technical approaches: 50% of studies that measured IB did not report how it was measured [37]. IB might have been measured by different methods	Future studies should: − Ensure reporting of how measurement was undertaken - Ensure uniformity in measurement technique for consistently comparable data across studies/countries
Testosterone		
Non-reporting	Three study did not examine any TT level as testicular function marker [10,38,44]; five studies (33.3%) assessed only pre-VR TT level [11,18,40,42,43]	Need for studies to consistently assess TT level as testicular function marker [81]. Related queries include: - What are the relationships between TT level and sperm parameters? - What are the relationships between TT level and pregnancy? - How accurate can TT level reflect deteriorated sperm parameters before VR? - In severe OAT, precision of pre-VR testosterone levels as early pre-operative predictors of VR outcomes?
Bidirectional effects	Most studies (71.4%) exhibited no change in TT after VR; two (*n* = 2, 28.6%) observed improvements	- What are the age, body mass index, varicocele grade, testicular volume, testicular blood flow characteristics of patients who are likely to have improved TT levels after VR? - Which of these or other characteristics, if any, exerts stronger effects on TT and can thus be used to predict this outcome?
Technical aspects	Technical approaches: 75% of studies that measured TT did not report how it was measured. TT might have been measured by different methods	Future studies should: − Ensure reporting of how measurement was undertaken - Ensure uniformity in measurement technique for consistently comparable data across studies/countries
Testicular Volume		
Non-reporting	Four studies (26.7%) did not assess any testicular volume; eight (53.3%) assessed only pre-VR testicular volume; only three studies (20%) assessed it both pre- and post-VR [34,36,40]	Testicular size significantly correlates with sperm density [86], smaller testicular volume correlate with decreased spermatogenesis [80]. Related queries include: - Needed: more studies that measure testicular volume before and after VR − Future studies should ensure reporting whether measurement was undertaken - What pre-VR testicular size cut-off is predictive of post-VR sperm count improvements? - In adolescents, post-VR catch-up growth is possible and notable [87] but remains unclear among adult men with severe OAT. What is the rate of post-VR catch-up growth? - How to affirm whether any defect in testicular size is due to varicocele or due to other causes?
Technical aspects	How was measurement undertaken? Of 11 studies that reported measuring testicular volume, three did not report how measurement was taken [9,11,38]; five (45.5%) reported measurement by USG [18,34,35,40,43], and three (27.3%) by orchidometer [13,36,37]	Future studies should: − Ensure reporting of how measurement was undertaken - Ensure uniformity in measurement technique for consistently comparable data across studies/countries
Genetic Considerations		
Underlying genetic or epigenetic factors	Four studies (26.7%) excluded patients with abnormal karyotypes or Y-microdeletion [9,18,42,43]; Nine (60%) did not report whether they excluded genetic abnormalities or otherwise	Future research should search for/appraise underlying genetic/epigenetic factors to help understand: - Heterogeneous clinical presentations [37]: Why men with non-severe grade I varicoceles have severe OAT [34]. Future prospective studies need to include genetic investigations (e.g. karyotyping, searching for Y-chromosome deletions) - Variable response to VR [37]: Why men with similar severity of OAT have different outcomes after VR in terms of deterioration, no improvement, or improvement. Can future prospective studies with genetic investigations provide better insights? VR among patients with genetic aberrations, related queries include: - Is VR in patients with genetic aberrations useful/recommended? [34] - How useful is VR in patients with genetic aberrations, particularly if they were to proceed to assisted pregnancy? [88] - What roles do microdeletions in the sequence of the L-type voltage-dependent calcium channel have in reducing pregnancy rate after VR? [88] - What role does TENT5D disruption play in the outcome of VR among OAT patients? [89]; and among severe OAT patients?
Economic appraisal/Cost-effectiveness	Only 3 (20%) studies had any economic evaluation: one compared financial costs, complications and pregnancy rates after sclerotherapy/other varicocelectomy techniques [35]; one compared cost-effectiveness of resulting pregnancy in VR for severe OAT vs direct to ICSI with no VR [43]; and one compared pregnancy rates of ART after VR vs ART with no prior VR and cost per pregnancy [11]	- What is the cost-effectiveness of achieving spontaneous or IUI pregnancy after VR in severe OAT? - Is rate of spontaneous pregnancy after VR for severe OAT high enough to support cost-effectiveness vs ART? - As live birth is the ultimate outcome, studies need to use live birth rate in calculating the cost-effectiveness - VR complications rate, severity and management should be included as cost components in economic appraisals VR versus no VR for severe OAT

ART: Assisted reproductive technology; FSH: Follicle stimulating hormone; IB: Inhibin B; ICSI: Intracytoplasmic sperm injection; IUI: Intra uterine insemination; IVF: In vitro fertilization; LBR: Live birth rate; LH: Luteinizing hormone; Morph: morphology; OAT: Oligoasthenoteratozoospermia; PR: Pregnancy rate; USG: Ultrasonography; Sp: Spontaneous pregnancy; T: Total; TT: Testosterone; VR: Varicocele repair.

Section B of Table 8 shows a range of research questions that were generated pertaining to VR for severe OAT, based on the knowledge gap that the current study unearthed. These questions would be essential and useful to enhance the evidence base of this topic, and the Table summarizes these ‘hotspots’ that are required to be addressed urgently for a more research-informed policy and practice.

## Discussion

Severe OAT, a challenge for male factor infertility [90], could be due to testicular failure, hormonal imbalance, varicocele, genetic disorders, infections, trauma, exposure to gonadotoxins, and idiopathic [91]. Although VR could result in better outcomes for severe OAT [18], reports indicate that VR does not result in consistent, same end-outcomes across patients.

To our knowledge, no previous report has undertaken an in-depth examination of the guidelines and published literature on the topic, analyzed it in terms of improvement, deterioration, or no significant change, and graded the evidence to draw conclusions premised on the number of studies and their level of evidence, highlighting the defects in the current research related to the topic and the way forward. The current report undertook these tasks.

Our main findings were: 1) The available guidelines do not address VR for severe OAT *per se*; rather, they deliver guidelines for VR for OAT. In this respect, the guidelines fully concur with each other in rather few instances; on more occasions, the guidelines intersect, creating only partial agreements, with recommendations unique to each individual guideline; 2) The published literature showed that the number of studies appraising VR in severe OAT were noticeably scarce. In addition, the literature mostly focused on basic sperm parameters, with much fewer studies examining changes in the wider range of hormones, testicular volume, pregnancy rate, or live birth rate as the ultimate fertility parameter; 3) Considering the number of studies, their risk of bias, and their level of evidence, the current literature leaves much to be desired; and 4) In terms of shortcomings, employing the limitations we observed across the guidelines and published literature, we mapped the current knowledge gaps and generated a raft of research questions to enhance the evidence base. Below, we discuss these findings in detail.

Pertaining to the guidelines, they fully agreed on only two aspects, namely recommending VR for palpable varicocele and abnormal semen; and conversely, not indicating it for subclinical/non palpable varicocele. While such agreements are useful, they fall short of consensus on guidance required for practitioners dealing with many clinical scenarios. Such situations included young males with progressive testicular failure [51,65] or reduced ipsilateral testicular volume [45,51]; and cases of isolated teratozoospermia [45] or azoospermia [67], unexplained infertility, or high sperm DNA fragmentation (SDF) [51]. In addition, discrepancies remain on the utility of evaluating related hormones in such cases, e.g. prolactin in OAT and hypogonadotropic hypogonadism [65]; as well as FSH and TT in oligozoospermia/OAT. Likewise, agreement on the value of e.g. karyotyping and Y chromosome microdeletion for severe OAT seems to remain under debate [65,67].

As for the literature, this review found that it mostly focused on assessments of the basic sperm parameters, concurring with that varicocele initially affects sperm motility, morphology, or both [92]. While earlier-stage varicocele might minimally impair sperm concentration, with advancement, the three parameters may be seriously compromised, resulting in severe OAT [93,94].

Post-VR improvements in sperm parameters in severe OAT do not necessarily directly translate to spontaneous pregnancy. The extent of the insult, i.e. pre-VR sperm concentration level is important, and a particularly critical group are those with ≤2 million sperm/ml. Others [13] found that for pre-VR concentrations ≤2 million/ml, the post-VR spontaneous pregnancy rate was lower than that of those with concentrations between 2 and 5 million/ml, suggesting that the former group will likely still require ART. Such constraints might explain why a recent global survey of clinicians reported that most would not undertake VR for patients with concentration <1 million/ml [95]. One probable cause for this might be Y chromosome microdeletions, which, despite their low incidence, can present with severe OAT/azoospermia [96], Sertoli cell-only syndrome, maturation arrest, or hypospermatogenesis, subject to the region affected [97]. Hence, such microdeletions need to be considered when weighing up severe OAT patients for VR. Types of Y chromosome microdeletions known to cause oligozoospermia up to azoospermia are deletions in the AZF region, with an incidence of one in 4000 men in the general adult population [98].

The 6^th^ edition of the WHO laboratory manual for the examination and processing of human semen [99] states the need for genetic evaluation following semen analysis in certain conditions but does not explicitly specify the semen parameters cutoff/s for performing genetic testing. The EAA and AUA/ASRM guidelines [65–67] provide finer details, acknowledging the need to assess genetic abnormalities for sperm concentrations ≤5 million/ml. The EAA and the European Molecular Genetics Quality Network (EMQN) best practice guidelines for molecular diagnosis of Y-chromosomal microdeletions [98] provide further fine grain recommendations, lowering this threshold to sperm concentrations <2 million/ml, based on the observation that clinically relevant deletions are found in azoospermia patients or severe oligozoospermia patients with sperm concentrations <2 million/ml. Moreover, a 2019 systematic review and meta-analysis suggested lowering this threshold further to ≤1 million/ml, due to the rare prevalence (0.8%) of Y chromosome microdeletions (AZF region deletions) among those with concentrations >1 million sperm/ml, and its higher prevalence (5%) among those with ≤1 million sperms/ml [100].

However, our findings might suggest otherwise (Table 9). When appraising the three studies included in the current review that undertook genetic assessment and reported the results, a different picture emerged. The Table depicts that, on the one hand, Najari et al. [41] included genetically normal patients and reported considerable improvement in post-VR concentration; while Addar et al. [18] and Majzoub et al. [9] also included genetically normal patients (excluded those with genetic abnormalities) but reported more modest post-VR improvements in sperm concentrations. This is despite that Najari’s [41] pre-VR concentration was critically lower than those reported by Addar et al. [18] and Majzoub et al. [9]. On the other hand, two studies included in the current review that did not report whether they undertook genetic assessment [11,38], both exhibited modest post-VR sperm concentration improvements; and a study that undertook genetic assessment for only a few patients but did not report the results [44] found higher post-VR improvements (even if one was to assume that some patients had genetic abnormalities). A point to note is that across these studies, despite improvements, the final post-VR sperm levels still face the challenge of a low probability of resulting in spontaneous pregnancy.

**Table 9.** Characteristics of studies reporting pre-VR sperm concentrations ≤2 million/ml (*N* = 7).

Study	Pre-VR MSC	Genetic assessment		Post-VR MSC
	(million/ml)	Undertaken?	Finding	(million/ml)
≤1 million sperm/ml				
Alkhayal 2024 [44]	0.25	Yes, but for few patients	NR	3.59
Poulakis 2006 [35]	0.4	Yes	NR	11.3
Najari 2019 [41]	0.85	Yes	Normal	12
1-2 million sperm/ml				
Dubin 2018 [11]	1.1	NR	—	5.37
Addar 2021 [18]	1.31	Yes	Excluded those with GA	5.23
Majzoub 2021 [9]	1.55	Yes	Excluded those with GA	7.5
Leung 2013 [38]	2	NR	—	6

For space considerations, only the first author is cited; GA: Genetic abnormalities; MSC: mean sperm concentrations; NR: not reported; VR: varicocele repair; —: no available data.

Collectively, these findings suggest that effects of genetic abnormalities require more understanding, as even in absence of such genetic abnormalities, as demonstrated across these three studies [9,18,41], the sperm concentration improvement rate was not uniform. Our findings support the notion that post-VR improvements might also be influenced by variables other than genetics (e.g. varicocele grade, extent of testicular damage, comorbidities, etc.). AZF screening is important before VR because such patients will most likely not benefit from VR in terms of sperm count [98], although VR can improve other parameters (e.g. SDF or OS), thus enabling an increase in pregnancy rate and live birth rate. This also highlights that studies need to control for potential confounders and stratify their analysis for more accurate results. Apart from two guidelines’ (EAA and EAU) [51,65] recommendations for karyotyping and PCR-based tests for Y chromosome microdeletions, more recent technology such as Next Generation Sequencing of multiple gene panels can be alternatives [101].

In connection with testicular volume, we observed that changes in this parameter were the least assessed in the included studies. It is not clear why this is the case. About 73.3% of the included studies examined testicular volume prior to VR, but only three (20%) re-examined it after VR [34,35,40]. Pre-VR assessment of testicular volume is warranted and concurs with the literature. For instance, a testicular atrophy index of ≥ 20% is more commonly observed in boys with varicocele [102]; patients with unilateral left varicocele and ipsilateral testicular atrophy have significantly worse semen analysis profiles compared to patients without atrophy [103]; and varicoceles can increase testicular atrophy as children progress through puberty [104]. Equally, post-VR testicular volume assessment is also necessary as adolescent varicocele with testicular hypotrophy is a common indication for surgery, where many of these patients may experience catch-up growth of the ipsilateral testis [105]; and 50–70% men will regain normal testicular growth after VR for testicular hypotrophy indication if they have clinically palpable varicoceles [87]. The samples of most of the studies included in the current review comprised adults, and the literature suggests that among adults, the effect of VR on testicular volume appears to be inconsistent, with reports of increased [32] or no significant change in testicular volume after VR [106], suggesting that the effect could be study dependent, despite that both studies observed an increase in total motile sperm count post-VR. Whilst hypotrophy suggests that testicular function could be reduced and spermatogenesis impaired, only two published guidelines (AUA/ASRM and EAU) [51,67] recommend undergoing VR for testicular hypotrophy. Nonetheless, some studies in the current review reported no change after VR suggesting that semen and hormonal changes are independent of testicular size [34,36].

Ultrasound is used to evaluate testicular parenchyma due to its accessibility, low cost and high sensitivity [107], although it has low specificity [108,109]. Recently, testis elastography has been employed to assess testis pathology in infertility and varicocele [110]; and mode B ultrasound and shear wave elastography can evaluate tissue stiffness and testicular volume in varicocele patients [111]. In all cases, the best practice recommendations on the imaging use of ultrasound need to be observed, such as the availability of an ultrasound device preferably with modern technology suitable for the cases and well-trained radiologists or ultrasound practitioners [112].

As for fertility-related outcomes, we noted that most of the included studies (86.7%) assessed the pregnancy rate, but much fewer (26.7%) assessed live birth rates. Although informative, pregnancy rates cannot be directly extrapolated to live births [113]. Unfortunately, the available guidelines [45,51,65–67] did not explicitly provide guidance about these outcomes post-VR.

In terms of hormonal evaluations (FSH, LH, IB, and TT), we noted that EAA’s guideline states that hormonal evaluation should be undertaken for all OAT patients (in agreement with AUA/ASRM) [65,67], and recommends VR for progressive testicular failure (in agreement with EAU) [51,65]. Despite this, some of our included studies did not evaluate any hormones [11,18,38–40,42,44], while others did not assess the full panel of hormones, sufficing with one [41], two [35] or three hormones [9,13,34,36]. These hormones provide insights pertaining to testicular damage pre-VR, and their levels might be prognostic of the likelihood of damage reversal post-VR [57,85,114]. Normal FSH, LH, and TT levels are required for normal spermatogenesis [80], and testicular damage can result in increased FSH/LH, decreased TT/IB, in addition to impaired basic sperm parameters [115]. FSH is negatively correlated with spermatogenesis [83]; IB is positively correlated [82] and is superior to FSH as a spermatogenesis indicator [83]. However, pertaining to FSH as a marker of testicular function, one of our included studies [35] noted that pre-VR FSH levels were not related to post-VR semen outcomes. Apart from a case report of one patient [41], the two studies that observed improvement in post VR hormonal parameters [34,35] used sclerotherapy as their method of VR, suggesting that the mode of VR employed might also play a role in improvement (intervention-dependent). Interestingly, although varicocele can lead to hypogonadism and VR can improve hormonal parameters, the hormonal levels can also be corrected by medication, such as human chorionic gonadotropins, aromatase inhibitors, or selective estrogen receptor modulators [116]. This is useful for varicocele patients with OAT who decline VR.

As for the knowledge gaps, four distinct types emerged. Linked to each gap, we outline potential avenues to push the boundaries of scientific work in this area. The first gap comprised a range of methodological limitations having to do with paucity of research, low level of evidence, study designs, narrow scale of research, modest sample sizes, lack of ultimate endpoint/s, and lack of consideration for confounding factors. Collectively, these can contribute to systematic bias introduced by study design [117], threaten internal validity [118]], and could represent frail evidence, missed/suboptimal findings, higher levels of uncertainty, non-consideration of confounding variables that might lead to misleading findings or ‘blurring’ or ‘contamination’ of effects, or decreased generalizability [71,119,120]. Future studies need to address these issues to generate high quality evidence.

The second gap had to do with the narrow research scope and measurement aspects. The former is exemplified by the lack of studies that adopted a broad range of assessments of all the relevant parameters required for comprehensive insights and understanding in cases of VR for severe OAT (sperm-, hormonal-, and fertility-related outcomes, as well as testicular volume), resulting in the frequent non-reporting of one/more parameter. The latter is illustrated in the issues encountered pertaining to the technical aspects and the technology employed when measuring a given parameter/s. In addition, for basic sperm parameters, there were further issues regarding the lack of consideration of variability and lack of serial follow ups. Collectively, these issues can affect the accuracy, reliability, consistency, and comparability of the data generated and potentially contribute to biased misjudgments when appraising the data. Hence, to provide evidence that deepens our understanding, future research needs to overcome such shortcomings to ensure that necessary measurements are all undertaken, and how each was undertaken, preferably ensuring uniformity in measurement techniques for consistently comparable data. A related gap is that outcomes are frequently not simply a clear cut ‘yes/no’, exemplified by our observed ‘multi-directional’ effects for sperm motility and morphology, as well as for some hormones e.g. FSH, IB, and TT (LH did not exhibit such effect). These phenomena provide fresh future opportunities to uncover any patient- and varicocele- related characteristics that might play significant roles in the improvement, no change or deterioration of these parameters, as well as the possibility of the use of any as pre-VR predictors of the outcome.

The third gap, the lack of research considering underlying genetic and epigenetic conditions when examining the effects of VR in severe OAT represented an area that could benefit from more investigation. While this has been discussed above (Table 9), the subsets of ‘non-responders’ where VR did not result in improvement, as well as the VR outcomes in severe OAT among genetically impaired populations require more exploration and further scrutiny. Hence, we support other calls for future research to specifically investigate the effect of VR in severe OAT due to genetic abnormality/ies to gain better understandings [34].

The final gap pertained to cost effectiveness. Even when clinical effectiveness appears to be confirmed, cost-effectiveness should be addressed in the current era of cost containment [121–123]. However, cost effectiveness assessments might be challenging due to differences of cost components across geographical regions, with direct/indirect costs, and out-of-pocket expense varying considerably across countries and insurance providers [121]. In addition, the ‘spontaneous pregnancy rate’ after VR that is frequently used in cost-effectiveness studies seems to be based on a likelihood/assumption of the potential of improved sperm parameters surpassing critical thresholds where spontaneous pregnancy becomes likely. Such assumption/s may represent misjudgments when the extent of the projected post-VR improvements is not as was initially expected, for instance, due to genetic abnormality/ies, or severe testicular failure. Future studies should address these issues, and for such special populations, it might be plausible that the outcome used in cost-effectiveness studies should also be adjusted, e.g. not utilize spontaneous pregnancy as the ultimate outcome but rather, other alternate outcomes e.g. pregnancy by intrauterine insemination or ART. In addition, economic evaluations will need to account for costs of the ART procedure, as well as costs incurred due to required medications for the male and the female partner due to e.g. hormonal imbalance or associated infections.

This review has limitations. The studies included were few, with high risk of bias and low level of evidence, resulting in insufficient high-quality data to draw firm conclusions. It would have been useful to have been able to address the causes of improvement of OAT in some studies and the reverse in the others, but unfortunately the studies did not provide explicit reasons for such findings or associated risk factors. Future studies should address these points. Despite this, the current study has many strengths. To our knowledge, this is the first in-depth assessment of outcomes of VR in severe OAT that employed two concurrent parallel methodologies, a scoping review of published guidelines and systematic review of the literature, to identify areas of agreements across the guidelines, and ascertain the evidence of the published studies and grade it, then collectively synthesize the information together to draw conclusions rooted in the available data on the topic, while highlighting the gaps and outlining a way forward for a ‘stronger’ evidence base.

## Conclusion

The current scoping review of published guidelines found that while there were guidelines pertaining to VR for OAT, no specific guidelines currently exist on VR for severe OAT per se. The systematic review revealed that VR results in multi-directional effects for reasons that remain to be uncovered. Hence, whilst VR does provide evident sperm parameters-related benefits to patients, infrequently VR leads to no significant change, and rarely detrimental sperm effects. In contrast, the hormonal parameters showed no significant change post-VR, but on rare occasions, some improvement. Pertaining to testicular volume, while the majority of patients consistently exhibited no significant change after VR, very few patients exhibited improvement. Notwithstanding, the limited number of available studies, their high risk of bias and low level of evidence resulted in insufficient and frail data for drawing solid conclusions. In bridging the identified raft of knowledge gaps and to advance the evidence base, future high quality, robust, large scale, multicentric, and multi-variable studies are required. Such inquiries need to simultaneously consider epi/genetic aspects, confounding and cost effectiveness that accounts for all male, female, and procedure-related cost components to further appraise the full range of outcomes of VR for this condition. In addition, further research on this topic should also pay attention to the extremely severe OAT subgroup of patients (sperm concentration < 1 million/ml).
